# Histopathology-correlated evaluation of magnetic resonance spectroscopy for high-grade glioma prediction: A small-scale neuro-oncology study with exploratory machine learning

**DOI:** 10.1016/j.ibneur.2026.09.007

**Published:** 2026-09-30

**Authors:** Nazli Sulaiman, Dost Muhammad, Azmat Ali, Malika Bendechache

**Affiliations:** a Department of Radiology, Ayub Teaching Hospital, Abbottabad, KPK, Pakistan; b CRT-AI, Data Science Institue, University of Galway, Galway, Ireland; c Department of Radiology, Ayub Teaching Hospital, Abottabad, KPK, Pakistan; d ADAPT Research Centre, School of Computer Science, University of Galway, Galway, Ireland

**Keywords:** Magnetic resonance spectroscopy, High-grade glioma, Neuro-oncology, Histopathology, Brain tumour, Diagnostic accuracy, Metabolic neuroimaging, Intracranial lesions, Glioma mimics, Machine learning

## Abstract

**Background:**

High-grade gliomas are aggressive primary brain tumours that require timely and accurate preoperative assessment. Magnetic resonance spectroscopy provides non-invasive metabolic information that may support neuro-oncological lesion characterisation. However, its tendency to show good sensitivity but limited specificity, particularly in the presence of metastasis, abscess, and other intracranial mimics, is already recognised. The clinical value of small, fully histopathology-confirmed cohorts lies in clarifying how these limitations appear in real diagnostic practice.

**Objective:**

This study evaluated the diagnostic performance of magnetic resonance spectroscopy for predicting high-grade glioma in a fully histopathology-confirmed cohort of patients with intracranial lesions. The study also aimed to characterise the false-positive pattern in clinically interpretable terms and to assess, as a secondary exploratory analysis, whether machine learning (ML) could add discriminatory value beyond the radiologist-derived magnetic resonance spectroscopy diagnosis.

**Methods:**

A retrospective cohort of 53 patients was analysed. All cases had histopathological confirmation, which was used as the reference standard. Patient-level variables included age, sex, magnetic resonance spectroscopy diagnosis, histopathological diagnosis, Cho/NAA, Cho/Cr, NAA/Cr, lactate, lipid, lesion location, enhancement pattern, necrosis, and oedema. Magnetic resonance spectroscopy was treated as the index test. Sensitivity, specificity, positive predictive value, negative predictive value, accuracy, F1-score, and balanced accuracy were calculated. Exploratory ML models were assessed using leave-one-out cross-validation.

**Results:**

The cohort included 31 males and 22 females, with a mean age of 48.1 ± 15.8 years. Histopathology confirmed high-grade glioma in 40 of 53 patients. Magnetic resonance spectroscopy classified 42 cases as high-grade glioma and 11 as non-high-grade glioma. Compared with histopathology, magnetic resonance spectroscopy achieved a sensitivity of 87.5%, specificity of 46.2%, positive predictive value of 83.3%, negative predictive value of 54.5%, accuracy of 77.4%, F1-score of 85.4%, and balanced accuracy of 66.8%. The seven false-positive cases were characterised as metastasis in three cases, abscess in three cases, and other non-high-grade pathology in one case. The best exploratory ML model was Random Forest with magnetic resonance spectroscopy diagnosis included, achieving an accuracy of 73.6% and an AUC of 0.646. However, this did not improve upon the diagnostic performance of radiologist-derived magnetic resonance spectroscopy diagnosis alone.

**Conclusion:**

In this small fully histopathology-confirmed neuro-oncology cohort, magnetic resonance spectroscopy demonstrated good sensitivity but limited specificity for predicting high-grade glioma. Rather than presenting this pattern as a new diagnostic phenomenon, this study contributes a clinically grounded confirmation with complete tissue verification, a clearly characterised false-positive mimic profile, and a transparent negative ML result. These findings support the use of magnetic resonance spectroscopy as an adjunctive metabolic neuroimaging tool and provide a cautious data point for future larger studies evaluating spectroscopy-guided and ML-supported diagnostic pathways.

## Graphical abstract

**Figure:**
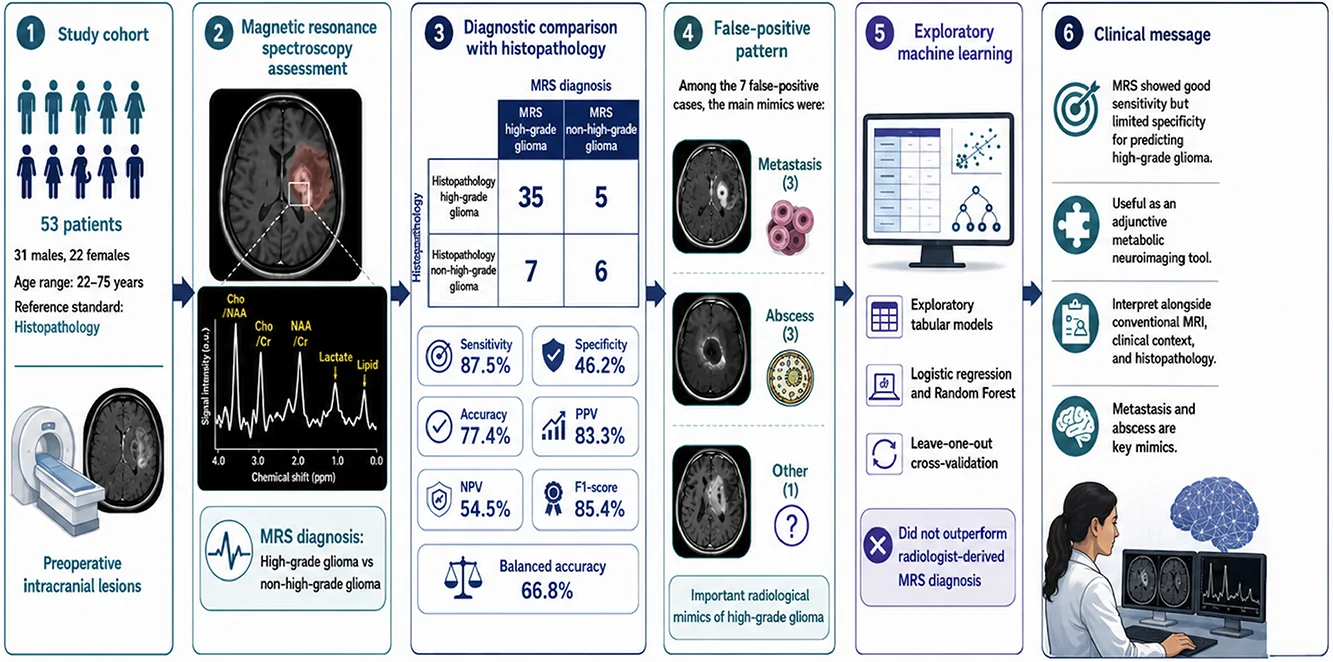

## 1 Introduction

Accurate preoperative characterisation of intracranial lesions is central to neuroradiological decision-making, neurosurgical planning, biopsy targeting, and patient counselling (Sogge et al., 2026). Among primary brain tumours, high-grade gliomas are clinically important because of their aggressive biological behaviour, infiltrative growth, and need for early multidisciplinary management (Javaid et al., 2026). The current World Health Organization classification of central nervous system tumours emphasises the integrated use of histopathological and molecular features, particularly in diffuse gliomas, while imaging remains the first-line tool for lesion detection, localisation, and preoperative assessment (Louis et al., 2021, Komori, 2022, Weller et al., 2021, Muhammad et al., 2024).

Conventional magnetic resonance imaging remains the foundation of brain tumour assessment. Features such as contrast enhancement, necrosis, peritumoural oedema, mass effect, and diffusion restriction can suggest aggressive pathology (Fan et al., 2026). However, morphological imaging may not always reliably distinguish high-grade glioma from metastasis, abscess, demyelination, low-grade glioma, or other tumour-mimicking conditions. This diagnostic overlap is particularly relevant in routine radiology practice because an imaging impression of high-grade glioma may influence the urgency of neurosurgical referral, biopsy approach, and treatment planning.

Magnetic resonance spectroscopy is an advanced magnetic resonance technique that provides non-invasive biochemical information from intracranial lesions (Pandey et al., 2026). Commonly evaluated metabolites include choline, creatine, N-acetylaspartate, lactate, and lipid peaks. High-grade gliomas often demonstrate increased choline-related ratios, reduced N-acetylaspartate, and variable lactate or lipid peaks, reflecting cellular proliferation, neuronal loss, necrosis, and anaerobic metabolism (Laino et al., 2020, Galijasevic et al., 2022, Guo et al., 2012). The Cho/NAA and Cho/Cr ratios are frequently used as supportive markers in glioma evaluation, although their diagnostic value depends on tumour heterogeneity, voxel placement, magnetic resonance acquisition parameters, spectral quality, and comparison tissue (Wang et al., 2017).

The diagnostic limitations of magnetic resonance spectroscopy are well recognised. Metabolic patterns may overlap between high-grade glioma, metastasis, abscess, lymphoma, inflammatory lesions, and necrotic tumours (Lai et al., 2008, Wang et al., 2017). Therefore, the observation that magnetic resonance spectroscopy may show good sensitivity but limited specificity is not, by itself, a new finding. Its practical relevance, however, remains important when assessed in local clinical cohorts where every case is verified against histopathology and where the exact nature of false-positive diagnoses can be examined.

The present study is positioned within this clinical and methodological context. Its contribution is not to claim that magnetic resonance spectroscopy limitations are newly discovered, but to provide a small-scale, histopathology-correlated neuro-oncology dataset in which all cases have pathological confirmation. Complete histopathological confirmation across the cohort is a major strength, particularly because diagnostic accuracy studies in neuroimaging may include mixed reference standards, imaging follow-up, or incomplete pathological verification. This design allows a direct comparison between the radiological magnetic resonance spectroscopy impression and the final tissue diagnosis.

A second contribution is the explicit characterisation of false-positive magnetic resonance spectroscopy findings. In the present cohort, false-positive cases were not treated merely as diagnostic errors, but were analysed as clinically meaningful mimics of high-grade glioma. The false-positive group consisted of metastasis, abscess, and other non-high-grade pathology, providing a clear radiological message for daily practice. Such an analysis is valuable because the clinical consequences of overcalling high-grade glioma differ from those of undercalling it, and because metastasis and abscess may require substantially different management pathways.

In parallel, machine learning (ML) has increasingly been explored in radiology and neuro-oncology (Qasem et al., 2026, Muhammad et al., 2025a, Muhammad et al., 2025b). Such models may combine clinical, imaging, and metabolic variables to support diagnostic classification. However, reliable artificial intelligence development requires adequate sample size, robust validation, transparent reporting, and assessment of clinical applicability. Guidelines such as CLAIM, STARD-AI, and TRIPOD+AI emphasise careful dataset description, validation design, reporting transparency, and avoidance of overinterpreting small exploratory models (Tejani et al., 2024, Sounderajah et al., 2025, Collins et al., 2024, Muhammad et al., 2026, Shah et al., 2026, Muhammad et al.). Therefore, in small single-centre datasets, ML should be treated as hypothesis-generating rather than clinically deployable.

The third contribution of this study is the transparent reporting of a negative exploratory ML result. Given the increasing enthusiasm for artificial intelligence in neuro-oncology, it is scientifically important to report when ML models do not add measurable value over radiologist-derived magnetic resonance spectroscopy diagnosis. In this cohort, exploratory ML was used as a secondary analysis to assess whether clinical, conventional imaging, and spectroscopy-derived variables could improve classification. The results are presented cautiously and are intended to inform future, larger studies rather than to support immediate clinical deployment.

Accordingly, this study evaluates the diagnostic performance of magnetic resonance spectroscopy for predicting high-grade glioma using histopathology as the reference standard. The primary aim is radiological and clinical: to quantify diagnostic accuracy and to examine false-positive and false-negative patterns in a fully histopathology-confirmed cohort. The secondary aim is methodological: to assess whether exploratory ML provides additional discriminatory value in a small tabular neuro-oncology dataset. By combining complete pathological verification, clinically interpretable false-positive analysis, and honest reporting of ML limitations, this study provides a cautious but useful contribution to small-scale neuro-oncology research.

**Table 1:**
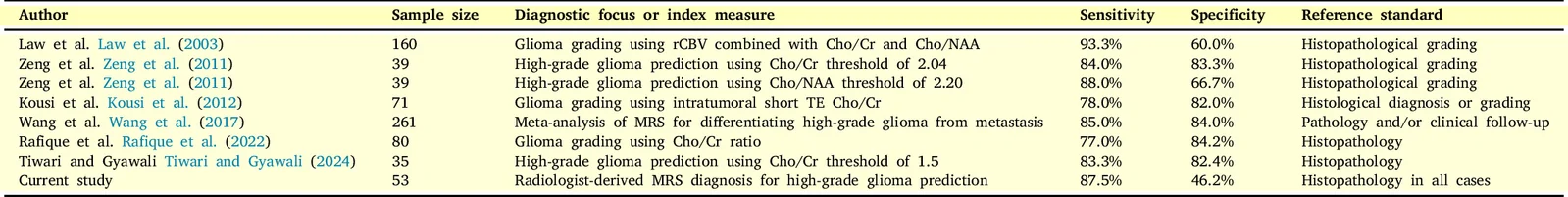
Selected previous MRS studies relevant to high-grade glioma assessment.

## 2 Literature context

Previous magnetic resonance spectroscopy studies have shown that metabolite-based assessment can support glioma grading and differentiation from mimicking intracranial lesions, but reported diagnostic performance varies across cohorts, acquisition protocols, metabolite thresholds, and reference standards. Table 1 summarises selected studies relevant to high-grade glioma assessment and places the present findings in context.

As shown in Table 1, the sensitivity observed in the present study is broadly consistent with previous MRS-based work. However, the specificity in the present cohort is lower than many published estimates. This difference is clinically informative rather than unexpected. The current cohort was fully histopathology-confirmed and included difficult non-high-grade mimics, particularly metastasis and abscess, which can show metabolic and morphological overlap with aggressive glioma. The lower specificity therefore supports the main clinical interpretation of this study: magnetic resonance spectroscopy is useful as an adjunctive metabolic imaging tool, but it should not be interpreted in isolation when clinically important mimics are present.

## 3 Materials and methods

### 3.1 Study design

This was a retrospective diagnostic accuracy study of patients with intracranial lesions who underwent magnetic resonance spectroscopy followed by histopathological confirmation. Magnetic resonance spectroscopy was considered the index test, and histopathology was considered the reference standard. The study was designed and reported in accordance with the principles of diagnostic accuracy reporting recommended by STARD (Bossuyt et al., 2015, Cohen et al., 2016).

### 3.2 Study population and case selection

The study cohort consisted of 53 patients with intracranial lesions who were evaluated at Ayub Teaching Hospital, Abbottabad, KPK, Pakistan. Informed consent was obtained from all patients before inclusion in the study. The cohort included 31 males and 22 females, giving a male-to-female ratio of approximately 1.4:1. Patient age ranged from 22 to 75 years, with a mean age of 48.1 ± 15.8 years and a median age of 44 years. Patients were identified retrospectively from the radiology and histopathology records of Ayub Teaching Hospital. Eligible cases were patients with intracranial lesions who underwent magnetic resonance spectroscopy and subsequently had histopathological confirmation. Cases were included if both the magnetic resonance spectroscopy diagnosis and the final histopathological diagnosis were available. Cases with incomplete imaging records, unavailable histopathological confirmation, or inadequate spectroscopy documentation were excluded. The patient selection process is summarised in Fig. 1.

Histopathological assessment was used as the reference standard for final diagnosis. The histopathological diagnoses included high-grade glioma, metastasis, abscess, low-grade glioma, demyelination, and other intracranial pathologies. The distribution of histopathological diagnoses is presented in Table 2. For the primary diagnostic accuracy analysis, the histopathological outcomes were dichotomised into high-grade glioma and non-high-grade glioma.

**Table 2:** Histopathological diagnoses in the study cohort.

Histopathological diagnosis	Number of cases
High-grade glioma	40
Metastasis	4
Abscess	4
Low-grade glioma	1
Demyelination	1
Other	3

Total	53

**Fig. 1:**
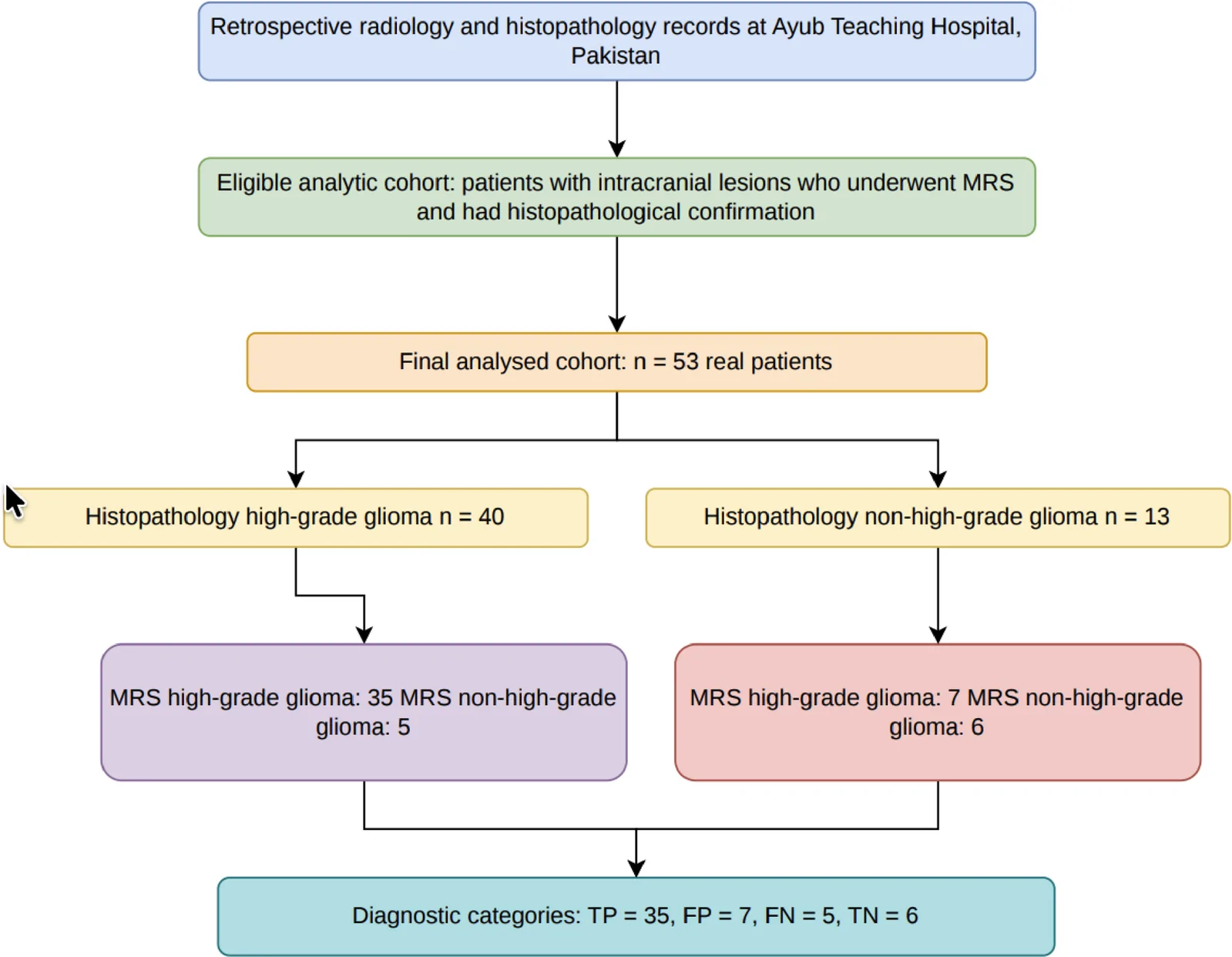
STARD-style patient flow diagram showing case identification, eligibility assessment, exclusion, and final inclusion in the diagnostic accuracy analysis.

### 3.3 Magnetic resonance imaging and spectroscopy acquisition

Magnetic resonance imaging and magnetic resonance spectroscopy examinations were performed using a [scanner vendor and model] system operating at [1.5T/3T]. Proton magnetic resonance spectroscopy was acquired using a [single-voxel/multivoxel] approach with a [PRESS/STEAM/chemical shift imaging] sequence. The acquisition parameters included echo time (TE) = [insert value] ms, repetition time (TR) = [insert value] ms, and voxel size = [insert value]. Water suppression was performed using [insert method], and spectral processing was performed using [insert software or workstation].

Voxel placement was performed by the reporting radiologist as part of routine clinical imaging assessment. Where feasible, the voxel was positioned within the solid enhancing or metabolically suspicious component of the lesion while avoiding haemorrhage, cystic change, extensive necrosis, bone, and cerebrospinal fluid contamination. The available spectroscopy-derived variables included Cho/NAA, Cho/Cr, NAA/Cr, lactate, and lipid. Lactate and lipid were recorded as present or absent.

Because this was a retrospective clinical dataset, some technical acquisition details were not consistently retrievable from the archived records. The unavailable parameters are acknowledged as a limitation because acquisition conditions can influence magnetic resonance spectroscopy interpretation.

### 3.4 Magnetic resonance spectroscopy diagnostic interpretation

Magnetic resonance spectroscopy diagnosis was recorded as either high-grade glioma or non-high-grade glioma. The diagnosis was treated as a radiologist-derived clinical interpretation rather than an automated threshold-based classification. The reporting radiologist integrated metabolite ratios, including Cho/NAA, Cho/Cr, and NAA/Cr, the presence or absence of lactate and lipid peaks, visual spectral pattern, and correlation with conventional magnetic resonance imaging features. These conventional imaging features included lesion location, enhancement pattern, necrosis, and oedema.

No single fixed metabolite threshold, such as Cho/NAA > 2 or Cho/Cr > 2, was used as an isolated decision rule for classifying a lesion as high-grade glioma. Instead, the final magnetic resonance spectroscopy impression reflected integrated radiological judgement in routine clinical practice.

Conventional imaging variables included lesion location, enhancement pattern, necrosis, and oedema. Enhancement was categorised as homogeneous, heterogeneous, or ring enhancement. Oedema was categorised as mild, moderate, or severe.

The following patient-level variables were included in the analysis:

• Demographic variables: age and sex.
• Spectroscopy-derived ratios: Cho/NAA, Cho/Cr, and NAA/Cr.
• Spectroscopy peak features: lactate and lipid.
• Conventional imaging features: lesion location, enhancement pattern, necrosis, and oedema.
• Radiological diagnosis: magnetic resonance spectroscopy impression.
• Reference diagnosis: histopathological diagnosis.

### 3.5 Reader assessment

Magnetic resonance spectroscopy interpretation was performed by [one/two] radiologist(s) with [insert number] years of experience in radiology/neuroradiology and brain tumour imaging. The magnetic resonance spectroscopy diagnosis used in the analysis corresponded to the clinical radiological impression recorded before final histopathological confirmation. The reporting radiologist was blinded to the final histopathological diagnosis at the time of initial imaging interpretation.

Inter-reader variability was not assessed because [insert reason, for example: the dataset was based on the final clinical report generated during routine radiology practice/only one reporting radiologist was available for the retrospective dataset]. This is acknowledged as a limitation of the study.

### 3.6 Reference standard

Histopathology was used as the reference standard. Cases were considered positive if histopathology confirmed high-grade glioma. All other histopathological diagnoses were considered non-high-grade glioma for the primary binary diagnostic accuracy analysis.

### 3.7 Diagnostic accuracy analysis

A two-by-two diagnostic table was constructed using histopathology as the reference standard. True positives were defined as cases labelled as high-grade glioma on magnetic resonance spectroscopy and confirmed as high-grade glioma on histopathology. False positives were defined as cases labelled as high-grade glioma on magnetic resonance spectroscopy but found to be non-high-grade glioma on histopathology. False negatives were cases labelled as non-high-grade glioma on magnetic resonance spectroscopy but confirmed as high-grade glioma on histopathology. True negatives were cases labelled as non-high-grade glioma on magnetic resonance spectroscopy and confirmed as non-high-grade glioma on histopathology.

The following diagnostic performance measures were calculated:

(1) $$Sensitivity=\frac{TP}{TP+FN}$$

(2) $$Specificity=\frac{TN}{TN+FP}$$

(3) $$PPV=\frac{TP}{TP+FP}$$

(4) $$NPV=\frac{TN}{TN+FN}$$

(5) $$Accuracy=\frac{TP+TN}{TP+TN+FP+FN}$$

(6) $$F1\text{-}score=\frac{2TP}{2TP+FP+FN}$$

(7) $$Balanced\;accuracy=\frac{Sensitivity+Specificity}{2}$$

95% confidence intervals were calculated for binomial proportions using the Wilson method.

### 3.8 Statistical analysis

Continuous variables were summarised using mean, standard deviation, median, and interquartile range. Categorical variables were summarised using frequencies and percentages. Differences between histopathologically confirmed high-grade glioma and non-high-grade glioma groups were explored using the Mann–Whitney U test for continuous variables. Fisher’s exact test or the chi-square test was used for categorical variables depending on the number of categories. All statistical comparisons were interpreted cautiously because of the limited sample size.

Because the feature-level comparisons were exploratory and intended to identify descriptive patterns rather than confirm definitive associations, no formal adjustment for multiple comparisons was applied. Therefore, p-values from these analyses should be interpreted as descriptive and hypothesis-generating rather than confirmatory.

No formal a priori sample size calculation was performed because this was a retrospective analysis of available histopathology-confirmed cases. Given the modest cohort size, the study may be underpowered to detect differences in individual metabolite ratios and categorical imaging features. Therefore, non-significant findings, particularly for Cho/NAA, Cho/Cr, NAA/Cr, lactate, lipid, necrosis, enhancement pattern, and lesion location, should not be interpreted as definitive evidence of no association.

For the exploratory machine learning analysis, receiver operating characteristic curves were generated for models that produced probabilistic outputs. Area under the receiver operating characteristic curve was calculated for penalised logistic regression and Random Forest models. These ROC analyses were used for visual and descriptive comparison only, because the machine learning component was exploratory and the cohort size was limited.

### 3.9 Exploratory machine learning analysis

A secondary exploratory ML analysis was performed using patient-level tabular variables. The aim was not to develop a deployable clinical artificial intelligence system, but to assess whether the available clinical, conventional imaging, and spectroscopy-derived variables contained additional discriminatory information beyond the radiological magnetic resonance spectroscopy diagnosis.

Two models were evaluated: penalised logistic regression (Greenwood et al., 2020) and Random Forest classification (Breiman, 2001). Two feature settings were considered. The first used age, sex, Cho/NAA, Cho/Cr, NAA/Cr, lactate, lipid, lesion location, enhancement, necrosis, and oedema, without including the radiological magnetic resonance spectroscopy diagnosis. The second used the same variables with the addition of magnetic resonance spectroscopy diagnosis.

Categorical variables were one-hot encoded and continuous variables were standardised where appropriate. To minimise information leakage, all preprocessing steps were performed within each leave-one-out cross-validation iteration. Standardisation was fitted only on the training portion of each fold and then applied to the held-out test case. One-hot encoding was fitted within the training fold and applied to the held-out case using the same feature mapping. The held-out test case was not used during preprocessing fitting, model training, or parameter estimation.

Leave-one-out cross-validation was used because the cohort contained only 53 patients. Model performance was reported using accuracy, sensitivity, specificity, positive predictive value, F1-score, balanced accuracy, and area under the receiver operating characteristic curve. The ML analysis was interpreted as exploratory, consistent with recommendations for transparent reporting of prediction and artificial intelligence studies in medicine (Tejani et al., 2024, Sounderajah et al., 2025, Collins et al., 2024, Lekadir et al., 2025).

Receiver operating characteristic curves were generated for the exploratory ML models that produced probabilistic outputs. These curves were used for visual and descriptive comparison of penalised logistic regression and Random Forest models.

The main hyperparameter settings used for the exploratory ML models are summarised in Table 3. No extensive hyperparameter optimisation was performed because of the limited sample size and exploratory nature of the analysis.

For Random Forest, feature importance was calculated to provide a descriptive ranking of the variables contributing to the model. Importance values from one-hot encoded categorical variables were grouped back to the corresponding original clinical variable where applicable. Because of the small and imbalanced dataset, feature importance was interpreted descriptively rather than as confirmatory evidence of biological or clinical importance.

**Table 3:** Hyperparameter settings used in the exploratory ML analysis.

Model	Hyperparameter settings
Penalised logistic regression	L2 regularisation; regularisation strength C=1.0; solver = lbfgs; maximum iterations = 1000; class weighting = none.
Random Forest	Number of trees = 500; splitting criterion = Gini impurity; maximum depth = none; minimum samples split = 2; minimum samples leaf = 1; bootstrap sampling = enabled; class weighting = none; random state = 42.

No deep learning model was trained because the dataset did not include raw imaging volumes or voxel-level spectroscopy data suitable for convolutional or transformer-based learning. The cohort size was also insufficient for reliable deep learning development and validation.

## 4 Results

### 4.1 Patient characteristics

The final cohort included 53 patients. The study population consisted of 31 males and 22 females, giving a male-to-female ratio of approximately 1.4:1. The mean age was 48.1 ± 15.8 years, with a median age of 44 years and an age range of 22 to 75 years. Histopathology confirmed high-grade glioma in 40 patients and non-high-grade glioma in 13 patients. The baseline demographic and histopathological characteristics of the cohort are summarised in Table 4.

**Table 4:** Baseline characteristics of the study cohort.

Characteristic	Value
Number of patients	53
Male	31
Female	22
Male-to-female ratio	1.4:1
Mean age	48.1 ± 15.8 years
Median age	44 years
Age range	22 to 75 years
Histopathological high-grade glioma	40
Histopathological non-high-grade glioma	13

### 4.2 Distribution of magnetic resonance spectroscopy diagnosis and histopathology

Magnetic resonance spectroscopy classified 42 patients as high-grade glioma and 11 patients as non-high-grade glioma. Histopathology confirmed high-grade glioma in 40 patients. The resulting diagnostic cross-tabulation is shown in Table 5.

Thus, the diagnostic categories were 35 true positives, 7 false positives, 5 false negatives, and 6 true negatives.

**Table 5:** Diagnostic cross-tabulation of magnetic resonance spectroscopy diagnosis against histopathology.

Histopathology	MRS high-grade glioma	MRS non-high-grade glioma	Total
High-grade glioma	35	5	40
Non-high-grade glioma	7	6	13

Total	42	11	53

### 4.3 Diagnostic performance of magnetic resonance spectroscopy

Using histopathology as the reference standard, magnetic resonance spectroscopy achieved good sensitivity but limited specificity for identifying high-grade glioma. The full diagnostic performance is presented in Table 6.

These findings indicate that magnetic resonance spectroscopy was more effective for identifying patients with high-grade glioma than for excluding the diagnosis. The positive predictive value was relatively high, while the negative predictive value remained limited.

**Table 6:** Diagnostic performance of magnetic resonance spectroscopy for identifying high-grade glioma.

Metric	Value	95% CI
Sensitivity	87.5%	73.9 to 94.5
Specificity	46.2%	23.2 to 70.9
Positive predictive value	83.3%	69.4 to 91.7
Negative predictive value	54.5%	28.0 to 78.7
Accuracy	77.4%	64.5 to 86.5
F1-score	85.4%	76.1 to 91.4
Balanced accuracy	66.8%	Not applicable

### 4.4 False-positive and false-negative patterns

Seven patients were classified as high-grade glioma on magnetic resonance spectroscopy but were found to have non-high-grade glioma on histopathology. The histopathological composition of these false-positive cases is presented in Table 7. Five patients were classified as non-high-grade glioma on magnetic resonance spectroscopy but were confirmed as high-grade glioma on histopathology.

As shown in Table 7, the false-positive group consisted mainly of metastasis and abscess, with three cases each, while one case was classified as other non-high-grade pathology. From a radiological viewpoint, this pattern is clinically important because metastases and abscesses may show ring enhancement, necrosis, oedema, lipid-lactate peaks, and altered choline-related ratios, which can overlap with aggressive glioma appearances. The false-negative cases are also clinically relevant because under-calling high-grade glioma may delay surgical planning, biopsy targeting, or oncological referral.

**Table 7:** Histopathological diagnoses among false-positive magnetic resonance spectroscopy cases.

Histopathological diagnosis	Number of false-positive cases
Metastasis	3
Abscess	3
Other	1

Total	7

### 4.5 Spectroscopy-derived variables

The distribution of age and spectroscopy-derived ratios according to histopathological high-grade glioma status is shown in Table 8. No statistically significant difference was observed between the high-grade glioma and non-high-grade glioma groups for Cho/NAA, Cho/Cr, or NAA/Cr.

Although the measured metabolite ratios did not differ significantly between the two groups, this should not be interpreted as evidence that spectroscopy lacks diagnostic value. The limited sample size, heterogeneous non-high-grade group, and likely overlap between malignant and inflammatory lesions may have reduced statistical power.

**Table 8:** Continuous variables according to histopathological high-grade glioma status.

Variable	High-grade glioma	Non-high-grade glioma	p-value
Age, median (IQR)	43.5 (34.0 to 60.5)	58.0 (37.0 to 61.0)	0.569
Cho/NAA, median (IQR)	2.87 (2.20 to 3.67)	2.96 (2.67 to 3.86)	0.463
Cho/Cr, median (IQR)	2.55 (1.60 to 3.13)	2.25 (1.91 to 2.78)	0.975
NAA/Cr, median (IQR)	1.31 (0.87 to 1.70)	1.50 (0.98 to 1.88)	0.414

### 4.6 Conventional imaging and spectroscopy peak features

The distribution of lactate, lipid, necrosis, oedema, enhancement pattern, and lesion location is summarised in Table 9. Oedema showed a near-significant trend, but this finding should be interpreted cautiously.

The overlap in lactate, lipid, enhancement pattern, necrosis, and oedema supports the clinical observation that high-grade glioma, metastasis, abscess, and other aggressive intracranial lesions may share similar imaging and metabolic features.

**Table 9:** Categorical imaging and spectroscopy features according to histopathological high-grade glioma status.

Variable	High-grade glioma	Non-high-grade glioma	p-value
Lactate present	23/40	4/13	0.119
Lipid present	22/40	8/13	0.756
Necrosis present	24/40	6/13	0.522
Oedema category	Mild 16, Moderate 10, Severe 14	Mild 1, Moderate 7, Severe 5	0.056
Enhancement pattern	Ring 15, Homogeneous 14, Heterogeneous 11	Ring 2, Homogeneous 6, Heterogeneous 5	0.331
Location	Frontal 5, Parietal 9, Temporal 10, Occipital 10, Basal ganglia 6	Frontal 0, Parietal 3, Temporal 3, Occipital 5, Basal ganglia 2	0.686

### 4.7 Exploratory machine learning results

Exploratory ML did not clearly improve upon the diagnostic performance of the radiological magnetic resonance spectroscopy diagnosis. The results are shown in Table 10.

Receiver operating characteristic curves for the exploratory ML models are shown in Fig. 2. The curves provide a visual comparison of penalised logistic regression and Random Forest models with and without the radiologist-derived magnetic resonance spectroscopy diagnosis. Among the ML models, Random Forest without magnetic resonance spectroscopy diagnosis produced the highest AUC of 0.700, while Random Forest with magnetic resonance spectroscopy diagnosis achieved an AUC of 0.646. These AUC values should be interpreted descriptively because the ML analysis was exploratory and the cohort size was limited.

**Table 10:** Exploratory ML performance using leave-one-out cross-validation.

Model	Feature set	Accuracy, 95% CI	Sensitivity	Specificity	PPV	F1-score	Balanced accuracy	AUC, 95% CI
MRS diagnosis alone	Radiological diagnosis	77.4% (64.5 to 86.5)	87.5%	46.2%	83.3%	85.4%	66.8%	Not applicable
Penalised logistic regression	Without MRS diagnosis	56.6% (43.3 to 69.0)	62.5%	38.5%	75.8%	68.5%	50.5%	0.527 (0.347 to 0.707)
Random Forest	Without MRS diagnosis	71.7% (58.4 to 82.0)	90.0%	15.4%	76.6%	82.8%	52.7%	0.700 (0.548 to 0.852)
Penalised logistic regression	With MRS diagnosis	62.3% (48.8 to 74.1)	67.5%	46.2%	79.4%	73.0%	56.8%	0.610 (0.440 to 0.780)
Random Forest	With MRS diagnosis	73.6% (60.4 to 83.6)	90.0%	23.1%	78.3%	83.7%	56.5%	0.646 (0.482 to 0.810)

CI, confidence interval; MRS, magnetic resonance spectroscopy; ML, machine learning; PPV, positive predictive value; AUC, area under the receiver operating characteristic curve. Accuracy confidence intervals were calculated using the Wilson method. AUC confidence intervals are presented descriptively. Formal statistical comparison against MRS diagnosis alone was not performed because the ML analysis was exploratory, the cohort size was limited, and the radiologist-derived MRS diagnosis was a binary clinical judgement rather than a probabilistic model output.

Random Forest feature importance ranking is shown in Fig. 3. The most influential variables were Cho/Cr, NAA/Cr, age, magnetic resonance spectroscopy diagnosis, oedema, and Cho/NAA. Lactate, enhancement pattern, lesion location, necrosis, sex, and lipid showed lower relative importance. These findings suggest that the model relied on a mixture of spectroscopy-derived ratios, demographic information, radiologist-derived magnetic resonance spectroscopy diagnosis, and conventional imaging features. However, the ranking should be interpreted cautiously because the dataset was small, imbalanced, and analysed using leave-one-out cross-validation.

**Fig. 2:**
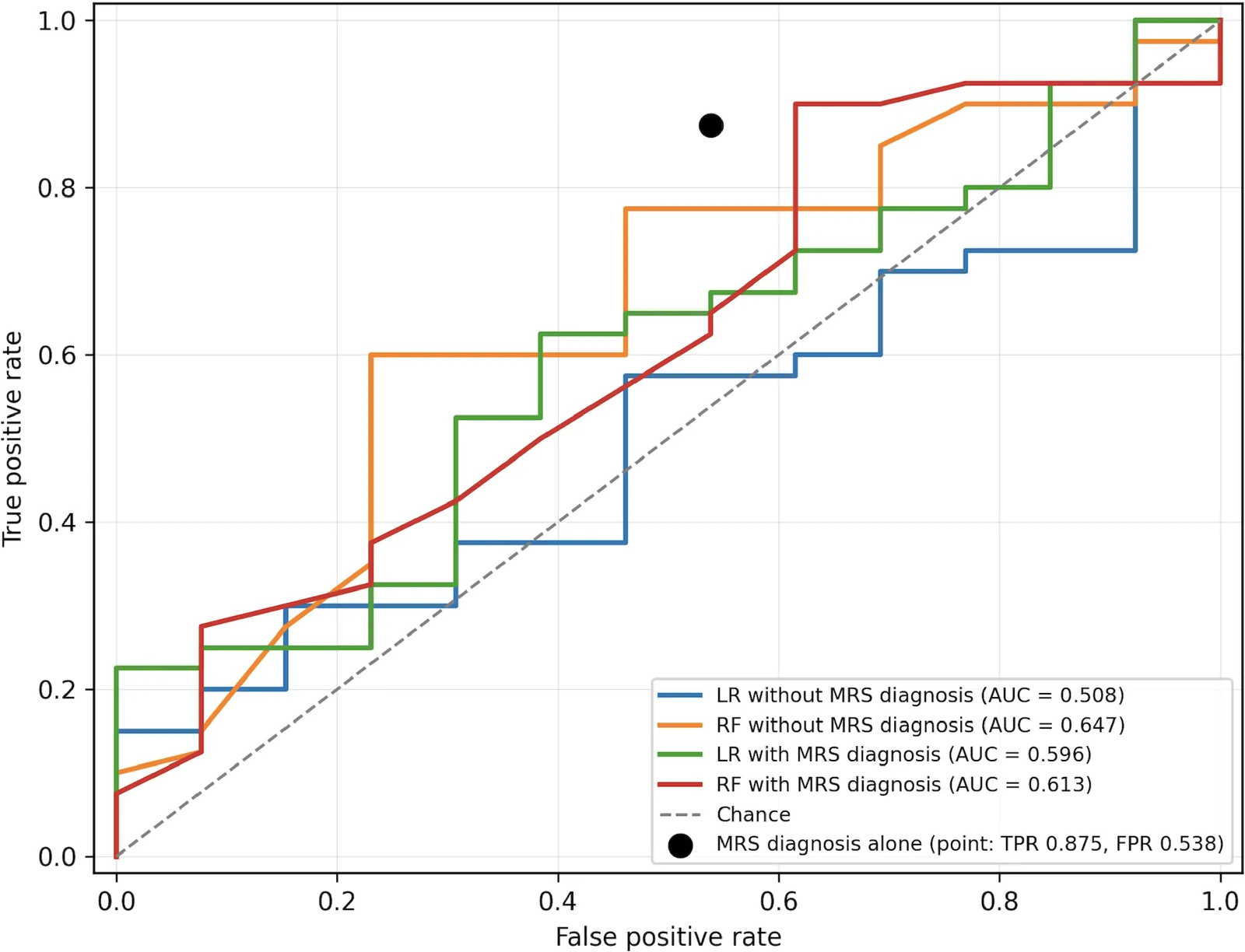
Receiver operating characteristic curves for exploratory ML models. The curves compare penalised logistic regression and Random Forest models with and without the radiologist-derived magnetic resonance spectroscopy diagnosis.

The ML models showed unstable performance, particularly in relation to specificity. Although the Random Forest models produced high sensitivity, they classified many non-high-grade cases as high-grade glioma. Notably, even when the radiologist-derived magnetic resonance spectroscopy diagnosis was included as an input feature, both ML models performed below magnetic resonance spectroscopy diagnosis alone in terms of overall accuracy. Penalised logistic regression with magnetic resonance spectroscopy diagnosis achieved an accuracy of 62.3%, while Random Forest with magnetic resonance spectroscopy diagnosis achieved an accuracy of 73.6%, compared with 77.4% for magnetic resonance spectroscopy diagnosis alone.

**Fig. 3:**
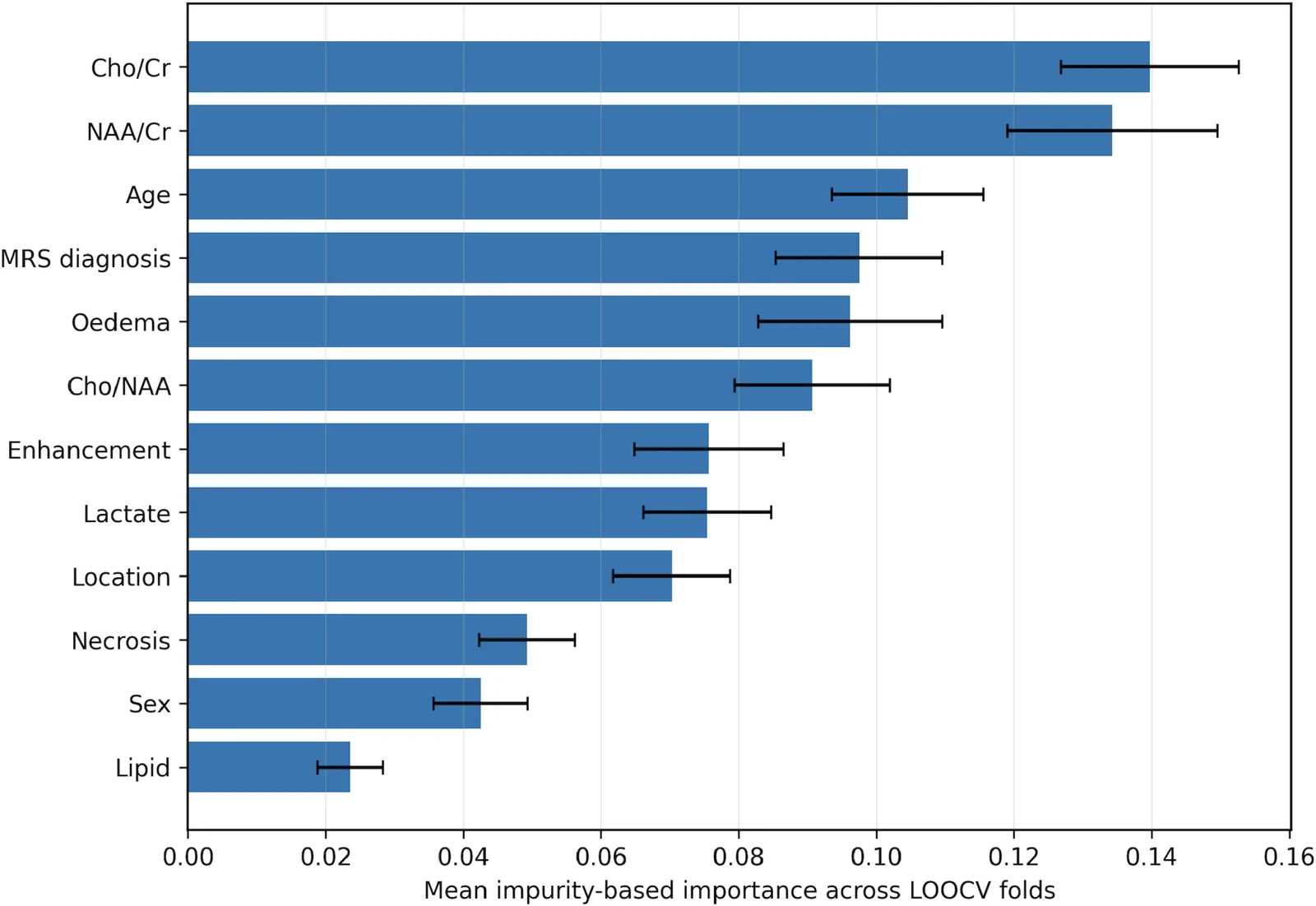
Random Forest feature importance ranking. Importance values are presented descriptively to show the relative contribution of patient-level clinical, spectroscopy-derived, imaging, and radiologist-derived variables in the exploratory ML analysis.

This finding suggests that the additional clinical, imaging, and spectroscopy-derived variables did not provide sufficient incremental discriminatory information in this small cohort. The lower accuracy of the ML models also reinforces the need to interpret exploratory ML results cautiously, particularly when the dataset is small, imbalanced, and clinically heterogeneous. In penalised logistic regression, the underperformance may partly reflect excessive coefficient shrinkage under regularisation, particularly when the strongest predictor was combined with multiple noisy or weakly informative variables. In Random Forest, performance may have been affected by high variance and unstable split selection under leave-one-out cross-validation, especially given the imbalanced sample size of 53 patients and only 13 non-high-grade glioma cases.

Therefore, the ML models may have diluted rather than strengthened the contribution of the single strongest predictor, namely the radiologist-derived magnetic resonance spectroscopy diagnosis. This pattern is clinically important because low specificity may increase false-positive referral, unnecessary anxiety, and potentially inappropriate diagnostic assumptions. Overall, the radiologist-derived magnetic resonance spectroscopy diagnosis remained the strongest and most clinically interpretable predictor in this dataset.

## 5 Discussion

This study evaluated the diagnostic performance of magnetic resonance spectroscopy for predicting high-grade glioma using histopathology as the reference standard. The principal finding is that magnetic resonance spectroscopy demonstrated good sensitivity but limited specificity. In clinical terms, spectroscopy identified most histopathologically confirmed high-grade gliomas, but it also overcalled several non-high-grade lesions as high-grade glioma.

### 5.1 Comparison with previous MRS studies

The sensitivity observed in the present cohort (87.5%) is broadly consistent with previous MRS-based studies of glioma assessment. Prior work has reported sensitivities ranging from approximately 77% to more than 90%, depending on the study design, metabolite ratio, imaging protocol, and reference standard (Law et al., 2003, Zeng et al., 2011, Kousi et al., 2012, Wang et al., 2017, Rafique et al., 2022, Tiwari and Gyawali, 2024). For example, Law et al. reported high sensitivity when perfusion MRI was combined with Cho/Cr and Cho/NAA, while Zeng et al. reported good sensitivity for Cho/Cr and Cho/NAA thresholds in glioma grading (Law et al., 2003, Zeng et al., 2011). Wang et al. also reported high pooled sensitivity for MRS in differentiating high-grade glioma from metastasis (Wang et al., 2017).

In contrast, the specificity in the present cohort (46.2%) was lower than many previous reports. This lower specificity is clinically informative rather than unexpected. Several previous studies focused on glioma grading or used more restricted comparison groups, whereas the present cohort included clinically difficult non-high-grade mimics, particularly metastasis and abscess. These lesions may show overlapping morphological and metabolic features with high-grade glioma, especially when ring enhancement, necrosis, oedema, lipid-lactate peaks, or elevated choline-related ratios are present. Therefore, the lower specificity in the present study likely reflects the real-world difficulty of distinguishing aggressive glioma from important mimics in a fully histopathology-confirmed cohort.

From the radiologist’s perspective, these findings support a balanced interpretation of spectroscopy. Magnetic resonance spectroscopy should not be regarded as a stand-alone diagnostic method. Instead, it should be integrated with lesion morphology, enhancement pattern, diffusion imaging, perfusion imaging, clinical history, immune status, evidence of systemic malignancy, inflammatory markers, and multidisciplinary discussion. Histopathology remains essential when imaging findings are equivocal or when treatment depends on precise tumour classification.

### 5.2 Biological explanation of false-positive findings

The false-positive cases are clinically instructive. In the present cohort, three false-positive cases were metastases, three were abscesses, and one was categorised as other. This pattern indicates that elevated metabolic suspicion on spectroscopy may reflect aggressive or destructive lesion biology rather than glioma-specific pathology.

The false-positive abscess cases can be explained by the metabolic features of infection, necrosis, and inflammatory tissue breakdown. Abscesses may demonstrate lactate peaks because of anaerobic glycolysis and hypoxic metabolism within the abscess cavity. Lipid peaks may arise from cellular membrane breakdown, necrotic debris, and inflammatory cell turnover. In some abscesses, additional metabolites such as amino acids, acetate, succinate, and alanine may be present, but these are not always reliably detected in routine clinical spectroscopy (Lai et al., 2008). As a result, an abscess with ring enhancement, oedema, necrosis, and lipid-lactate peaks may resemble a necrotic high-grade glioma on metabolic imaging.

The false-positive metastasis cases are also biologically plausible. Metastases may demonstrate elevated choline because of increased cellular membrane turnover and tumour proliferation. Necrotic metastases may also show lipid and lactate peaks, reflecting cell death, hypoxia, and necrotic tumour components. These metabolic features overlap with high-grade glioma, particularly glioblastoma, which may also show elevated choline, reduced N-acetylaspartate, necrosis, and lipid-lactate components (Wang et al., 2017). Therefore, MRS may detect aggressive lesion biology without being specific for glioma lineage.

The false-negative cases are equally important. Five histopathologically confirmed high-grade gliomas were labelled as non-high-grade glioma on magnetic resonance spectroscopy. Possible explanations include tumour heterogeneity, voxel placement error, sampling of less aggressive tumour regions, necrotic or cystic components, spectral quality limitations, and overlap in metabolite ratios. This reinforces the need for careful voxel placement and correlation with conventional magnetic resonance imaging.

The metabolite ratio analysis did not demonstrate statistically significant differences between high-grade glioma and non-high-grade glioma groups. This finding should be interpreted cautiously. The dataset was small, and the non-high-grade group was heterogeneous. In addition, metastasis and abscess may share metabolic features with aggressive glioma, particularly when necrosis and lipid-lactate peaks are present. The absence of statistical significance in this cohort therefore does not negate the broader clinical value of spectroscopy, but it does emphasise the importance of contextual interpretation.

### 5.3 Exploratory machine learning interpretation

The exploratory ML analysis did not improve diagnostic performance beyond the radiological magnetic resonance spectroscopy diagnosis. This result is not unexpected. The cohort was small, imbalanced, and heterogeneous, with only 53 patients and 13 non-high-grade cases. ML models in this setting are highly vulnerable to overfitting and unstable specificity. Although Random Forest achieved high sensitivity, its specificity was low, meaning that it tended to classify many non-high-grade cases as high-grade glioma. Such behaviour would be clinically unsafe if used without radiologist oversight.

Importantly, this study does not support the use of deep learning on the current dataset. Deep learning would require raw imaging data, adequate sample size, appropriate segmentation or region-of-interest definition, independent validation, and transparent reporting. The current dataset is more suitable for diagnostic accuracy analysis and exploratory tabular ML. Future artificial intelligence work should follow reporting frameworks such as CLAIM, STARD-AI, and TRIPOD+AI, particularly if the model is intended for diagnostic decision support (Tejani et al., 2024, Sounderajah et al., 2025, Collins et al., 2024).

### 5.4 Relevance to modern molecular neuro-oncology

The findings should also be interpreted in the context of the modern WHO classification of central nervous system tumours. The 2021 WHO classification emphasises integrated diagnosis combining histopathological and molecular information, particularly in diffuse gliomas (Louis et al., 2021, Komori, 2022, Weller et al., 2021). Markers such as IDH mutation status, 1p/19q co-deletion, MGMT promoter methylation, and Ki-67 proliferation index have important diagnostic, prognostic, and therapeutic relevance.

The present study used histopathological high-grade glioma status as the reference standard, but molecular markers were not available in the analysed dataset. This limits the ability to align MRS findings with modern molecular tumour subtypes. For example, IDH-mutant astrocytoma, IDH-wildtype glioblastoma, and oligodendroglioma with IDH mutation and 1p/19q co-deletion may have different biological behaviour, prognosis, and treatment pathways. Future studies integrating MRS, conventional MRI, histopathology, and molecular markers may provide a more precise framework for evaluating metabolic imaging in neuro-oncology.

Overall, the findings support the practical role of magnetic resonance spectroscopy as part of a multiparametric neuroradiological assessment. Its good sensitivity may help raise suspicion for high-grade glioma, but its limited specificity means that radiologists should carefully consider metastasis, abscess, and other mimics, particularly when spectroscopy findings are not fully concordant with conventional imaging or clinical information.

## 6 Clinical implications

The findings have several implications for radiology practice. First, magnetic resonance spectroscopy may be useful when conventional magnetic resonance imaging findings are suspicious but not definitive for high-grade glioma. A high-grade spectroscopy impression may support urgent neurosurgical referral, multidisciplinary review, and biopsy planning.

Second, false-positive diagnoses are clinically important. In this cohort, metastases and abscesses accounted for most false-positive cases. These findings emphasise that lipid-lactate peaks, elevated choline-related ratios, ring enhancement, and necrosis should be interpreted in clinical and multiparametric imaging context rather than as glioma-specific findings.

Third, a non-high-grade spectroscopy impression should not be used to exclude high-grade glioma. The negative predictive value was only 54.5%, and five high-grade gliomas were undercalled by spectroscopy.

Fourth, exploratory ML did not provide clear added value in the present small dataset. Future artificial intelligence studies would require larger multicentre datasets, raw imaging data, standardised acquisition protocols, external validation, and close radiological supervision.

## 7 Limitations

This study has several limitations. First, it was retrospective, single-centre, and based on a modest cohort of 53 patients. Although complete histopathological confirmation strengthens the analysis, the small sample size limited statistical power and subgroup evaluation.

Second, the dataset was imbalanced, with 40 high-grade glioma cases and 13 non-high-grade glioma cases. This may have affected specificity estimates and contributed to instability in the exploratory ML analysis.

Third, the non-high-grade glioma group was heterogeneous, including metastasis, abscess, low-grade glioma, demyelination, and other diagnoses. This reflects real-world diagnostic complexity but limits disease-specific comparison between individual mimics.

Fourth, molecular markers such as IDH mutation status, MGMT promoter methylation, 1p/19q co-deletion, and Ki-67 index were not available. Therefore, MRS findings could not be interpreted in relation to contemporary molecular tumour subtypes.

Finally, external validation was not performed. The findings should therefore be interpreted as single-centre, hypothesis-generating evidence. The exploratory ML analysis was based on tabular variables only and should not be considered evidence for a deployable artificial intelligence system.

## 8 Future work

Future studies should extend this work through larger, prospective, multicentre cohorts with standardised magnetic resonance imaging and spectroscopy protocols. In addition to the variables analysed in the present study, future datasets should include scanner field strength, spectroscopy sequence, echo time, repetition time, voxel placement strategy, spectral quality indicators, and radiologist experience. These variables would allow a more detailed evaluation of how acquisition parameters and interpretative expertise influence magnetic resonance spectroscopy performance in differentiating high-grade glioma from clinically important mimics such as metastasis and abscess.

Future work should also incorporate raw magnetic resonance imaging and spectroscopy files, where available. This would support more advanced radiomics, deep learning, and spatially informed modelling approaches, while allowing direct assessment of lesion morphology, voxel localisation, and metabolic heterogeneity. However, such approaches should be developed only with adequate sample size, external validation, and transparent reporting.

Finally, future studies should include molecular tumour markers such as IDH mutation status, MGMT promoter methylation, 1p/19q co-deletion, and Ki-67 index. These markers are central to modern neuro-oncology and may improve integrated diagnostic modelling when combined with conventional imaging, magnetic resonance spectroscopy, histopathology, and carefully validated ML methods.

## 9 Conclusion

Magnetic resonance spectroscopy demonstrated good sensitivity but limited specificity for predicting high-grade glioma in this histopathology-correlated cohort. The results support the use of spectroscopy as an adjunctive neuroradiological tool, particularly for raising suspicion of high-grade glioma, but not as a stand-alone diagnostic method. False-positive cases involving metastasis and abscess highlight the need for integrated interpretation with conventional magnetic resonance imaging, clinical context, and histopathological confirmation.

The exploratory ML analysis did not outperform the radiological magnetic resonance spectroscopy diagnosis and should be considered hypothesis-generating only. Larger prospective studies with standardised imaging protocols, raw imaging data, molecular markers, and external validation are needed before artificial intelligence-based decision support can be recommended for clinical use.

## CRediT authorship contribution statement

**Nazli Sulaiman:** Writing – original draft, Visualization, Validation, Methodology, Investigation, Formal analysis, Data curation, Conceptualization. **Dost Muhammad:** Writing – original draft, Validation, Software, Resources, Methodology, Funding acquisition, Formal analysis, Data curation, Conceptualization. **Azmat Ali:** Writing – review & editing, Validation, Supervision, Project administration, Investigation, Formal analysis, Conceptualization. **Malika Bendechache:** Writing – review & editing, Validation, Supervision, Resources, Project administration, Investigation, Funding acquisition, Formal analysis, Conceptualization.

## Ethics statement

Ethical approval for this study was obtained from the review board/ ethics committee of the Ayub Medical College Abbbottabad prior to conducting this study. All necessary patient consent procedures were completed in accordance with institutional requirements.

## Funding

This research was partially supported by Taighde Éireann – Research Ireland under grant numbers GOIPG/2025/8471, 18/CRT/6223 (RI Centre for Research Training in Artificial Intelligence), 13/RC/2106/ P_2_ (ADAPT Centre) and 13/RC/2094/P_2_ (Lero Centre). For the purpose of Open Access, the authors has applied a CC BY public copyright licence to any Author Accepted Manuscript version arising from this submission.

## Declaration of competing interest

The authors declare that they have no known competing financial interests or personal relationships that could have appeared to influence the work reported in this paper.

## Data Availability

The data used in this study are not publicly available due to institutional and ethical restrictions. However, they may be made available for research purposes upon reasonable request to the corresponding author, subject to the necessary institutional approval and applicable ethical requirements.
